# Multi-region hemispheric specialization differentiates human from nonhuman primate brain function

**DOI:** 10.1007/s00429-013-0620-9

**Published:** 2013-08-09

**Authors:** Hsiao-Ying Wey, Kimberley A. Phillips, D. Reese McKay, Angela R. Laird, Peter Kochunov, M. Duff Davis, David C. Glahn, Timothy Q. Duong, Peter T. Fox

**Affiliations:** 1 Research Imaging Institute, University of Texas Health Science Center, San Antonio, TX 78229 USA; 2 Department of Radiology, University of Texas Health Science Center, San Antonio, TX 78229 USA; 3 Athinoula A Martinos Center for Biomedical Imaging, Department of Radiology, Massachusetts General Hospital, Boston, MA 02129 USA; 4 Department of Psychology, Trinity University, 1 Trinity Place, San Antonio, TX 78212 USA; 5 Southwest National Primate Research Center, Texas Biomedical Research Institute, San Antonio, TX USA; 6 Department of Physics, Florida International University, Miami, FL USA; 7 Maryland Psychiatric Research Center, University of Maryland, Baltimore, MD 21201 USA; 8 Olin Neuropsychiatry Research Center, Institute of Living, Yale University, New Haven, CT USA; 9 Department of Psychiatry, Yale University, New Haven, CT 06511 USA; 10 Department of Pharmacology, University of Texas Health Science Center, San Antonio, TX 78229 USA

**Keywords:** Brain evolution, Brain connectivity, Functional connectivity, Resting-state networks, Primates, fMRI

## Abstract

The human behavioral repertoire greatly exceeds that of nonhuman primates. Anatomical specializations of the human brain include an enlarged neocortex and prefrontal cortex (Semendeferi et al. in Am J Phys Anthropol 114:224–241, 2001), but regional enlargements alone cannot account for these vast functional differences. Hemispheric specialization has long believed to be a major contributing factor to such distinctive human characteristics as motor dominance, attentional control and language. Yet structural cerebral asymmetries, documented in both humans and some nonhuman primate species, are relatively minor compared to behavioral lateralization. Identifying the mechanisms that underlie these functional differences remains a goal of considerable interest. Here, we investigate the intrinsic connectivity networks in four primate species (humans, chimpanzees, baboons, and capuchin monkeys) using resting-state fMRI to evaluate the intra- and inter- hemispheric coherences of spontaneous BOLD fluctuation. All three nonhuman primate species displayed lateralized functional networks that were strikingly similar to those observed in humans. However, only humans had multi-region lateralized networks, which provide fronto-parietal connectivity. Our results indicate that this pattern of within-hemisphere connectivity distinguishes humans from nonhuman primates.

## Introduction

Hemispheric specialization refers to the differential functions of the left and right cerebral hemispheres. One of the most pronounced behavioral asymmetries in humans is hand dominance, with a majority of individuals expressing right-handedness. Though the neuroanatomical differences underlying this major behavioral specialization are minor (Amunts et al. 1996), motor-task functional activation studies using fMRI readily illustrate this behavior neurophysiologically (Biswal et al. 1995). Similarly, left-lateralized language dominance is the rule in humans, to the degree that left hemisphere lesions routinely yield aphasia, while right hemisphere lesions do so only rarely. As in the manual motor system, lateralized neuroanatomical correlates that subserve these functional differences are relatively modest (Geschwind and Levitsky 1968; Steinmetz 1996) while neurophysiological metrics acquired during speech paradigms mirror the behavioral asymmetry (Petersen et al. 1988; Fox et al. 2000). Finally, attentional dominance is a strongly lateralized function in humans, with hemi-spatial neglect occurring with right hemisphere lesions but not with left hemisphere lesions (Mesulam 1981). To our knowledge, there are no such anatomical asymmetries yet discovered that underlie this behavioral specialization, but functional imaging studies show this effect quite readily (Fox et al. 2006). Collectively, these observations illustrate that humans show extreme behavioral lateralization, which parallels task activation as imaged by fMRI. Neurobiological explanations of these marked brain-behavioral asymmetries should be sought with functional, rather than structural, imaging methods.

Nonhuman primates (NHP) also demonstrate hemispheric specialization. As in humans, the most marked expression of this is seen in hand dominance. Chimpanzees, baboons, and capuchin monkeys display hand dominance for various skilled motor tasks, though at levels of lateralization that are less pronounced than humans (Hopkins 2007). Anatomical asymmetries related to hand dominance have been reported in the motor cortices of great apes (Hopkins and Cantalupo 2004; Gannon et al. 1998; Hopkins et al. 2010) and monkeys (Phillips and Sherwood 2005; Phillips and Thompson 2013). Behavior and lesion studies indicate that monkeys, like humans, preferentially use the auditory system in the left hemisphere to process vocalizations (Heffner and Heffner 1984; Poremba et al. 2004), though again at levels less lateralized than humans. In addition, some indications suggest functional lateralization for the production of attention-getting vocalizations in chimpanzees which is associated with asymmetry of language area homologs (Taglialatela et al. 2008). To our knowledge there are no data on lateralization of attention in NHP. Thus, there appears to have been positive selection within the primate order for increasing complexity of hemispheric specialization.

Task-activation networks in humans correspond to intrinsic functional connectivity networks (ICNs) (Biswal et al. 1995; Fox and Raichle 2007; Smith et al. 2009). Functional connectivity can be inferred from spontaneous BOLD signal fluctuations arising from low frequency (<0.1 Hz) brain activity (Biswal et al. 1995; Damoiseaux et al. 2006). These ICNs provide a means of identifying the neurophysiological underpinnings of the brain’s functional architecture, which in some cases reflects the underlying structural connectivity of the brain (van den Heuvel et al. 2009) without task-engagement. Furthermore, resting-state connectivity networks are robust in sleeping infants and anesthesia, hence this connectivity is intrinsic and can be examined in anesthetized NHP (Fransson et al. 2007; Vincent et al. 2007).

In the present study, we used resting-state functional imaging to examine the evolution of lateralized ICNs in representative primate species: humans, chimpanzees (a Great Ape), baboons (an Old World Primate), and capuchin monkeys (a New World Primate). We expected to find evidence for continuity of lateralized ICNs with increasing complexity within the primate order. However, if hemispheric specialization is unique to humans, as has been proposed (Crow 1998), then lateralized ICNs associated with motor function, attention, and especially language should only appear in humans.

## Methods

### Subjects

We acquired 100 resting-state fMRI scans from human participants (45 males, 55 females; age = 43.2 ± 12.1 years), five resting-state fMRI scans from chimpanzees (*Pan troglodytes*, 1 male, 4 females; age = 24.8 ± 12.5 years), 24 resting-state fMRI scans from baboons (*Papio hamadryas* spp., 14 females; age = 12.7 ± 4.5 years) and 25 resting-state fMRI scans from capuchin monkeys (*Cebus apella*, 3 males, 5 females; age = 9.2 ± 7.9 years). Human imaging data were provided by the Genetics of Brain Structure and Function study (structural MRI—Kochunov and Davis 2010; resting-state fMRI—Glahn et al. 2010). Humans were instructed to relax with eyes open during scans. Nonhuman primates were anesthetized with isoflurane (1–2 %) for the purpose of restraint and to keep the subjects immobilized during the collection of the brain images. Subjects remained anesthetized throughout the MRI procedure while a veterinarian continually monitored respiration rate, heart rate, and oxygen consumption. The Institutional Review Board and Institutional Animal Care and Use Committee of the University of Texas Health Science Center at San Antonio and/or the Texas Biomedical Research Institute (San Antonio, Texas) approved the research.

### Image acquisition

All MRI studies were performed on a 3T Siemens TIM TRIO. Gradient echo EPI was used for BOLD resting-state images with the following parameters: TR/TE = 3000/30 ms. In NHPs, images were acquired with matrix = 124 × 124, field of view (FOV) = 12.4 × 12.4 cm (1 × 1 × 1.9 mm resolution), and 27 slices for 30 min; in human studies, images were acquired with matrix = 128 × 128, FOV = 22 × 22 cm (1.7 × 1.7 × 3 mm resolution), and 43 slices for 8.5 min. Each subject underwent high-resolution T1-weighted 3D Turbo-flash imaging with an adiabatic inversion contrast pulse and the following parameters: TE/TR/TI = 3.04/2100/785 ms, flip angle = 13°, and 800 micrometer isotropic voxel resolution. These images were subjected to retrospective motion correction to achieve optimal gray/white matter contrast (Kochunov and Davis 2010).

### Image pre-processing

Analyses were performed using The Oxford Center for Functional Magnetic Resonance Imaging (FMRIB) software, FSL (www.fmrin.ox.ac.uk/fsl). Standard image pre-processing for functional MRI data was employed. Time-series data were skull-stripped using automated brain extraction software (Smith 2002) and motion corrected (Jenkinson et al. 2002). Images were then band-passed temporal filtered at 0.01–0.08 Hz (fslmaths). The resulting data were spatial smoothed with either a 6 mm FWHM (capuchin monkeys and baboons) or an 8 mm FWHM (chimpanzees and humans) Gaussian kernel. Individual time-series data were then registered to its own high-resolution T1-weighting anatomical image and further registered to a standard template brain. Both co-registration steps use affine linear registration with 12 degrees-of-freedom. Human data were co-registered to an MNI-152 atlas brain, and data of NHPs were co-registered to brain study specific templates created by averaging anatomic images of each species.

### Independent component analysis

Intrinsic connectivity networks (ICNs) were derived using temporal-concatenation independent component analysis (Beckmann and Smith 2004), which is a well-established and robust data-driven group-level functional connectivity approach (Smith et al. 2009). To facilitate comparison of results herein with previous publications and to compare ICNs across species, the dimensionality of all ICA analyses was 20. The identification of ICNs was based on visual inspection of spatial similarity. In addition, to confirm the results were not biased due to difference in sample sizes (one hundred (8.5 min) datasets in human vs. five to twenty-five (30 min) datasets in NHPs), the same functional connectivity analysis using TC-ICA was applied for a subset of human subjects (*N* = 18, 18 (8.5 min) data were chose to resemble 5 (30 min) worth of data in NHPs). Spatially similar ICNs were found in this subset of data.

### Lateralization index

Lateralization index (LI) was calculated using equation: *LI* = *(Left* – *Right)/(Left* + *Right),* where *Left* and *Right* represents voxel counts from the region-of-interests (ROIs) defined within the left and right hemisphere of each ICN (*Z* > 3), respectively. LI was derived from all primate species and plotted in Fig. 4. A LI larger than 0 is considered left-lateralized, while a LI <0 is considered right-lateralized. A |LI| ≥ 0.2 is typically considered as strongly lateralized.

### Homotopic connectivity analysis

Studies have proposed that the strength of homotopic connectivity as an index for the tendency of hemispherical asymmetry (Zuo et al. 2010). Homotopic connectivity (termed voxel-mirrored homotopic connectivity in early studies) was calculated between every pair of symmetric inter-hemispheric voxels. Specifically, we calculated the Pearson’s correlation coefficient between the time series of each voxel and its symmetric inter-hemispheric voxel. The resulting correlation coefficients were then Fisher z-transformed. Individual z-maps representing the strength of homotopic connectivity were averaged by subject within each group and normalized by dividing each voxel by the average hemisphere value.

## Results

**Fig. 1:**
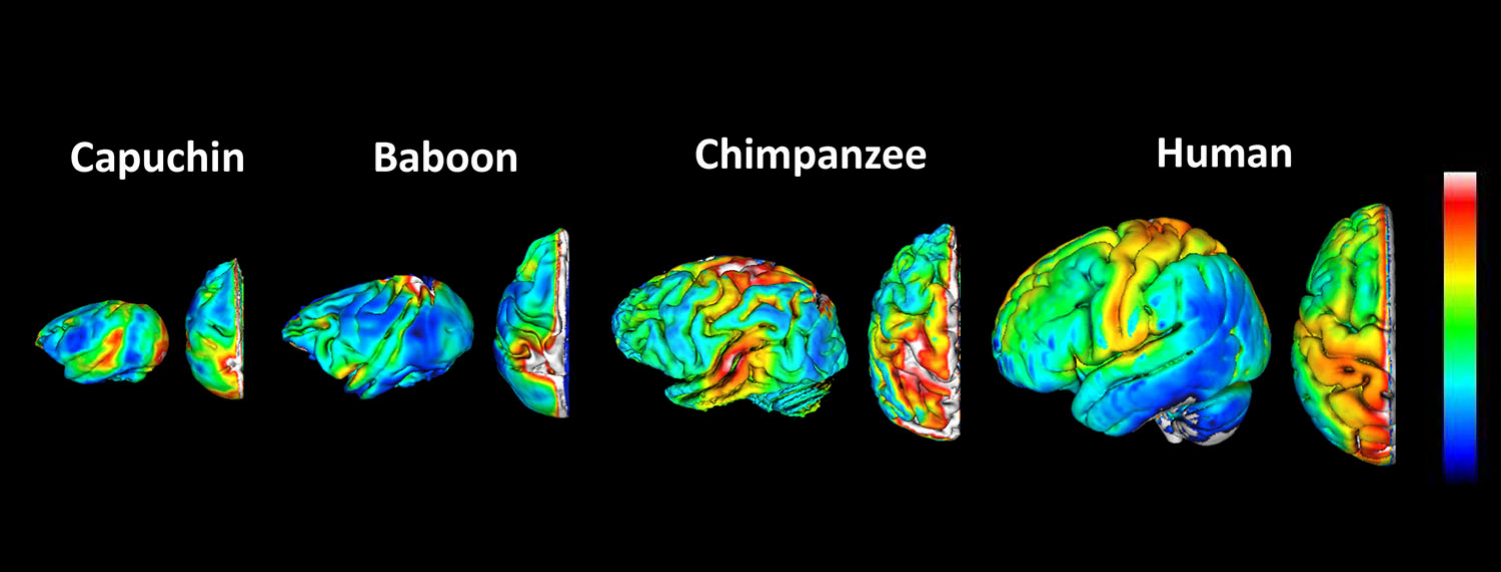
Inter-hemispherical synchrony of spontaneous brain activity. The strength of inter-hemispherical coherences is shown. The higher degree of coherence indicates a higher inter-hemispherical connectivity and coordination. The correspondence of the maps is proof-of-concept that the resting-state data contributing to the findings herein is broadly similar appropriate for cross-species comparison

We computed the homotopic functional coherences and demonstrated similar spatial patterns among humans, chimpanzees, baboons, and capuchin monkeys (Fig. 1). This suggests that the overall distributions of functional connectivity are similar across some primate species (Stark et al. 2008). Furthermore, resting-state based functional connectivity in humans and anesthetized NHP is broadly consistent and appropriate for finer grained analyses of specific networks (Rilling et al. 2007; Hutchison et al. 2011).

**Fig. 2:**
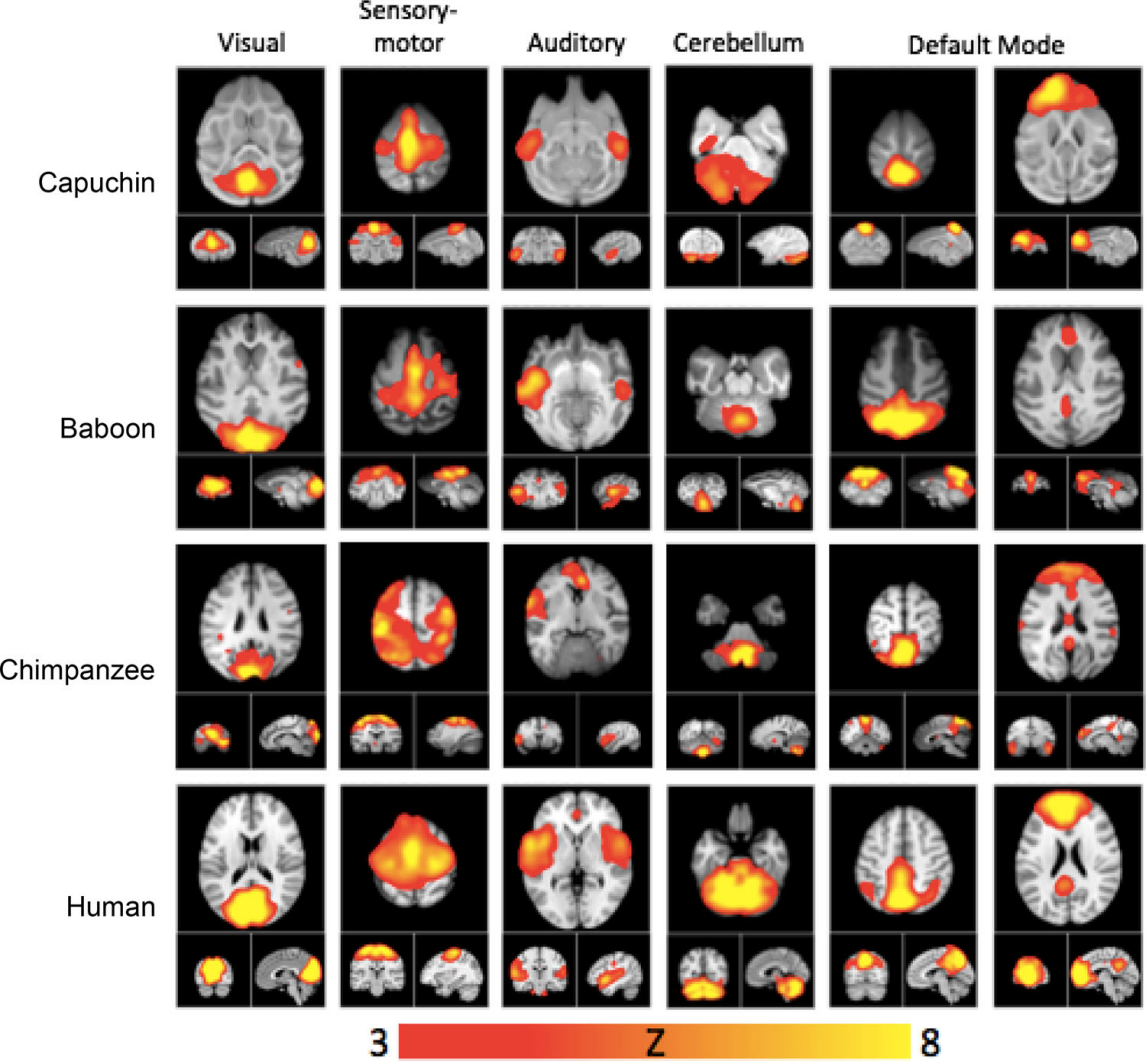
Bilateral intrinsic connectivity networks across primate species. ICNs from the four primate species that correspond to basic bottom-up processing. The visual, sensory-motor, auditory, and cerebellar networks incorporate the input of information from the surround and the output of motor plans. The default mode network is associated with self-referential, non-directed processing. These ICNs were symmetric and most similar across species

Most of the ICNs, in both humans and NHP, were not lateralized. Several spatially similar ICNs, albeit with minor differences, were found across primate species and pertained to basic sensory-motor functions (Fig. 2). Visual and somatosensory ICNs, including the occipital cortex and pre- and post-central sulcus, respectively, were robust in all primate species. In addition, the default mode network (DMN)—which encompasses the anterior and posterior cingulate cortex, the medial and lateral superior parietal lobe, and the medial prefrontal cortex—was spatially similar in all species. Furthermore, each group demonstrated a strikingly similar split of the DMN into two separate anterior and posterior components (Fig. 2).

**Fig. 3:**
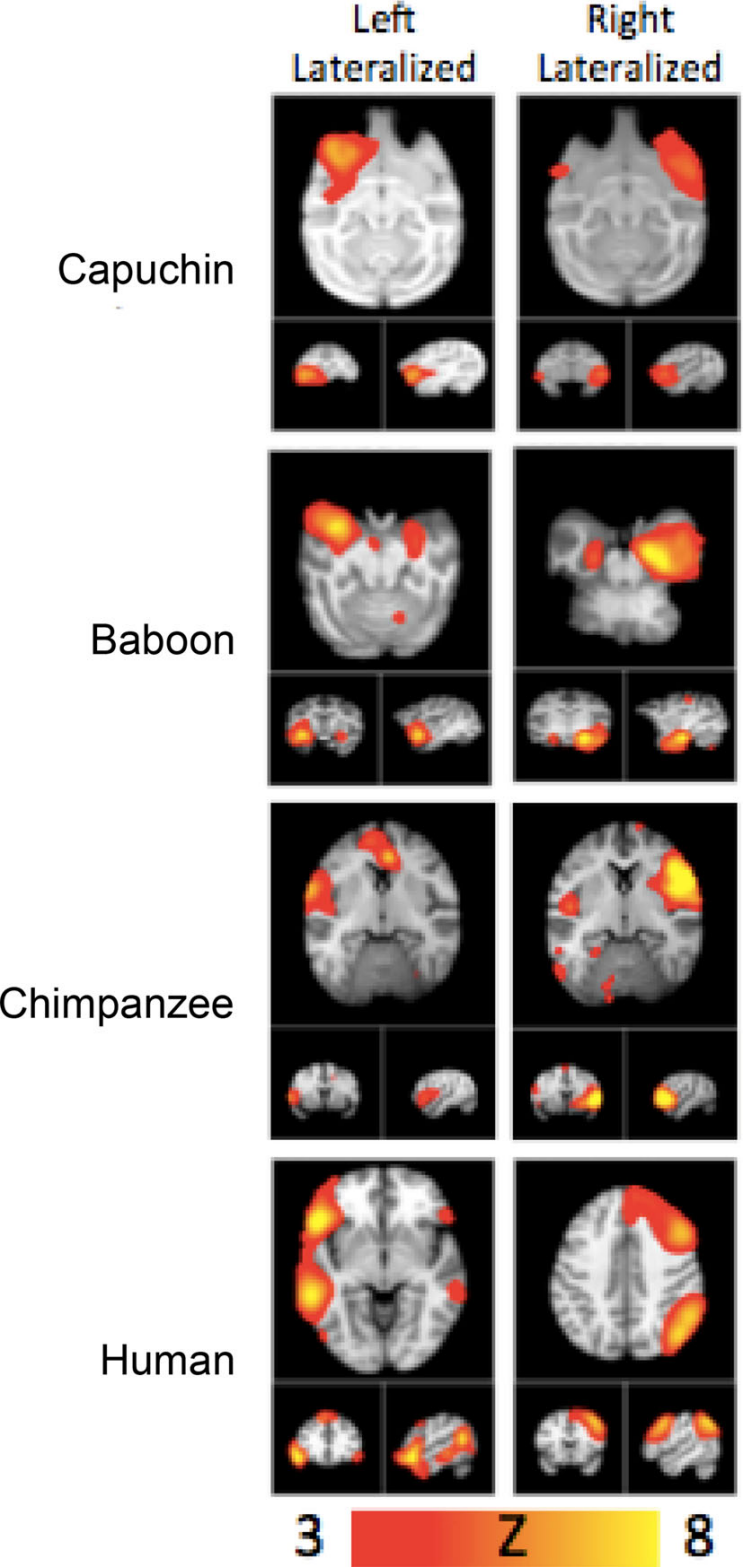
Unilateral intrinsic connectivity networks across primate species. Left- and right-lateralized ICNs that correspond top-down cognitive functionality. In humans, the left lateralized fronto-parietal network is associated with speech and language processing, while the right lateralized fronto-parietal network is associated with reasoning, attention, inhibition and working memory. These networks are confined to a single frontal node in non-human primates

**Fig. 4:**
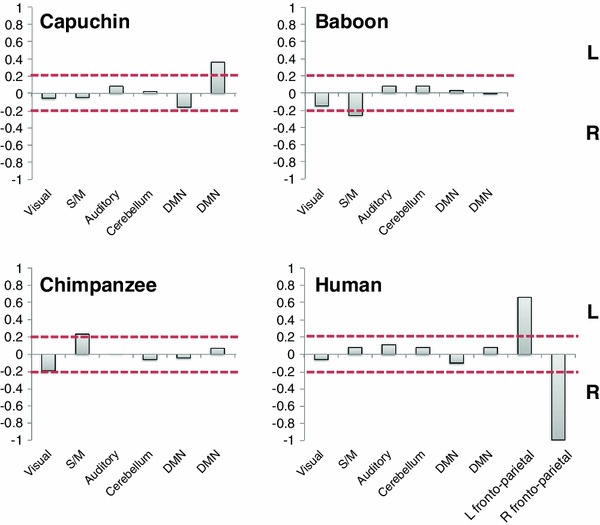
Lateralization Indices (LI). Lateralization indices (LI) for ICNs illustrated in Figs. 2 and 3. ICNs are considered lateralized when the LI is larger than 0.2 or lower than −0.2. While separate frontal and parietal networks were lateralized in all four groups (not shown), the fronto-parietal human networks were the only multi-region lateralized ICNs in any of the species

We found the left- and right- asymmetric ICNs generally reported in humans to be present across species. Contrary to expectation, these ICNs were strikingly similar in all species, suggesting that functional laterality emerges early in the primate lineage (Fig. 3). Laterality indices indicated significant lateralization of these ICNs in all species (Fig. 4). This strongly argues against hemispheric specialization as a unique feature of humans. However, an unexpected and notable difference existed in humans, where two strongly left-lateralized and one right-lateralized ICN were consistently shown to include frontal and parietal components (Damoiseaux et al. 2006; Smith et al. 2009). In NHP, the frontal and parietal components were split into separate lateralized ICNs.

## Discussion

Our results provide compelling evidence that functional laterality is phylogenetically conserved in primates. As in humans, functional laterality is far more profound than anatomical asymmetry. Furthermore, the notable difference in functional laterality between humans and NHP is fronto-parietal connectivity, where multi-region (inter-lobar) networks existed only in humans. While functional laterality alone is not a distinguishing characteristic of human brains, we suggest the observed pattern of multi-region functional connectivity is. Passingham (2008) postulated that it is the connections of neurons, particularly those in association cortices, that account for the distinguishing mental characteristics of humans.

When not engaged in goal-directed behavior, spontaneous fluctuations in brain activity give rise to coherent and structured ICNs that are nearly identical to networks engaged during cognitive or behavioral tasks (Smith et al. 2009; Biswal et al. 2010). Given this high degree of correspondence between rest and task, the behavioral correlates of human networks are reliable and have been assessed using previously published ICA of task-based data (See Laird et al. 2011 for detailed descriptions of each human network). In contrast, the default mode putatively supports self-referential or non-directed cognitive processing (Gusnard et al. 2001) and served as a benchmark in two regards for the current report. First, NHP subjects were anesthetized and humans were not. It is possible that the lateralized multi-region hemispheric, inter-lobar connectivity is present in NHPs yet masked by anesthesia. However, as the DMN—which has inter-lobar connectivity—was readily and uniformly detectable in all species, it was deemed unlikely that a lateralized functional network with inter-lobar connectivity would be masked by anesthesia. Second, consistency of default mode networks across species was used to affirm the validity of making group comparisons in groups of varying size. Because these default mode based benchmarks were met, implications of task-associated networks were assessed.

ICNs underlying basic input of sensory information from the surround and output of simple motor plans were extremely homogenous across humans and NHPs. The visual ICN was strongly linked to simple visual stimuli such as flashing checkerboards. This sensorimotor ICN was associated with action and somesthesis corresponding to hand movements, including tasks such as finger tapping, grasping, pointing, and electrical and vibrotactile stimulation. The auditory ICN was related to audition, including tone and pitch discrimination tasks. The cerebellar ICN was associated with action and somesthesis, including both overt and covert object recognition even though no other language or speech tasks were associated with this region. Indeed, the cross-species congruence of ICNs and behavioral repertoire in bottom-up processing is clear.

In contrast, human ICNs responsible for higher aspects of cognition (attention and language) required inter-lobar fronto-parietal connectivity, while NHP ICNs included only the frontal subcomponent. The left-lateralized fronto-parietal network strongly mapped to a host of semantic, phonologic, and orthographic language tasks such as word generation and covert reading, as well as working and explicit memory tasks, such as paired associate recall, cued encoding and recognition. The right-lateralized fronto-parietal ICN was associated with multiple cognitive processes, such as reasoning, inhibition, and memory. Moreover, top-down processing that is thought to be exclusive to humans evidently requires intrinsic connectivity of multiple brain regions.

Because our findings ultimately reflect connectivity, white matter tracts are surely involved. The superior longitudinal fasciculus, a group of white matter fibers located in the frontal, parietal and temporal regions provides connectivity within the fronto-parietal networks. Recent diffusion tensor imaging (DTI) and dissection studies revealed three different components of this perisylvian tract, which connect to specific cortical areas within the frontal, parietal and temporal lobes (Martino et al. 2012). In humans, this tract and working memory performance share genetic influence (Karlsgodt et al. 2010). Thiebaut de Schotten et al. (2012) reported major differences in the arcuate fasciculus, the inferior fronto-occipital fasciculus, and the inferior aspect of the superior longitudinal fasciculus. Another DTI study revealed differences across humans, chimpanzees, and macaques in the arcuate fasciculus, the white matter tract connecting the frontal and temporal cortices, which is associated with language in humans (Rilling et al. 2008). Our results are in strong support of their overall premise, that similarities suggest preserved functions across anthropoids while connectivity differences indicate human specific functional specialization. We build upon these findings by demonstrating that the observed structural connectivity has multi-regional, lateralized physiological properties and is correlated with human specific behavior.
